# Changes in Ovaries and Uterus after Aglepristone Administration in the Third Week of Luteal Phase of Non-Pregnant Bitches

**DOI:** 10.1371/journal.pone.0121597

**Published:** 2015-03-27

**Authors:** Kamil Kacprzak, Piotr Jurka, Izabella Dolka, Michał Czopowicz, Anna Ruszczak, Anna Duszewska

**Affiliations:** 1 Department of Small Animal Diseases with Clinic, Faculty of Veterinary Medicine, Warsaw University of Life Sciences, Warsaw, Poland; 2 Department of Pathology and Veterinary Diagnostics, Faculty of Veterinary Medicine, Warsaw University of Life Sciences, Warsaw, Poland; 3 Laboratory of Veterinary Epidemiology and Economics, Faculty of Veterinary Medicine, Warsaw University of Life Sciences, Warsaw, Poland; 4 Department of Morphological Sciences, Faculty of Veterinary Medicine, Warsaw University of Life Sciences, Warsaw, Poland

## Abstract

**Objective:**

The mechanism of aglepristone action in the placentation time in the bitch remains unclear. The aim of this study was to describe the mechanism by which aglepristone influences ovaries and uterus and to measure the levels of steroid sex hormones in non-pregnant bitches.

**Materials and Methods:**

Fourteen bitches assigned to a study (n=9) and control (n=5) group were given aglepristone and saline solution, respectively, on the 19th and 20th day after LH peak. On the 26th day after LH peak an ovariohysterectomy was performed. Blood samples were screened for estradiol and progesterone concentrations. Ovaries and uterine horns and bodies were isolated for histological and morphometrical diagnosis and immunohistochemistry analysis of α-estrogen and progesterone receptor expression.

**Results:**

A decrease of progesterone (p<0.01) and no differences in total estrogen level in the study group were observed. There were no significant differences either in the histomorphometry or α-estrogen and progesterone receptors expression in ovaries. Increase in expression of progesterone receptors in endometrium without surface epithelium of horns (p<0.05), endometrial surface epithelium (p<0.05), myometrium of uterine body (p<0.01) and estrogen receptors in endometrium without surface epithelium of horns (p<0.05) was observed. Elevated estrogen receptors probably increased sensitivity of tissues to estrogens in the bloodstream and led to notable inflammation, haemorrhages, and hyperplasia in endometrium with mononuclear immune cell infiltration. The myometrium of horns and endometrium of uterine body of study bitches were significantly thicker than in the control group (p<0.05 and p<0.01). Furthermore myometrium of uterine body was thicker than myometrium of horns (p<0.001) and expression of progesterone receptors was higher in uterine body (p<0.01). No differences were observed between endometrium of horns and body within groups.

**Conclusion:**

To the knowledge of the authors this is the first study, which describes the inflammatory effect developing in uterus in response to aglepristone administration, and attempts to elucidate its mechanisms.

## Introduction

Aglepristone (RU534) is a progesterone receptor (PR) antagonist used for termination of pregnancy from early to late luteal phase of the ovarian cycle. However, the exact physiologic consequence of blocking of nuclear PR in uterus and ovaries remains unclear. Histological findings following the use of RU534 after placentation (between 25th and 45th day of pregnancy) revealed the accumulation of secretions within glandular chambers and degenerative lesions in the tissue. Moreover, endometrium was significantly thicker than in pregnant, untreated bitches [1]. Aglepristone given between the 25th and 45th day of the luteal phase does not seem to influence PR and α-estrogen receptor (ERα) density in uterus [1]. Nevertheless, there is no information available to the authors about those parameters in the placentation time–the 19th to 21st day after the luteinizing hormone (LH) peak. This is a time of various prominent changes in the reproductive system of both pregnant and non-pregnant bitches. During this period structure of uterine wall is completely reorganised mainly under progesterone (P4) influence. In pregnant bitches relaxin blood level begins to increase and is accompanied by LH level growth [2].

The aim of this study was to determine the influence of RU534 administered to non-pregnant bitches in the 19th and 20th day of the luteal phase on microscopic structure of uterine wall, expression of PR and ERα in uterine wall and in ovaries as well as steroid hormone levels in blood.

## Materials and Methods

### Animals

Fourteen mixed-breed bitches, 2–5 years of age and 10–26 kg bw, healthy on the basis of routine clinical examination were enrolled in the study. According to the medical history of each dog, the interestrus intervals of previous cycles were normal. During the study, animals stayed in the Small Animal Hospital of the Faculty of Veterinary Medicine, and were fed with commercial diets at will. Since the first appearance of vulvar serosanguineous discharges indicating the onset of proestrus, vaginal smears had been collected daily from each bitch and promptly examined upon hematoxylin-eosin staining (H-E) until the first day of cytological diestrus. The protocols involving the care and use of animals for these experiments were approved by the Bioethical Committee of the Warsaw University of Life Sciences. This research was supported by the National Science Centre, Poland grant No. N N308 566940. Investigations were carried out after obtaining the agreement from the Third Local Animal Experimentation Committee at the Warsaw University of Life Sciences and the agreement of the Dean of the Faculty of Veterinary Medicine, Warsaw University of Life Sciences. Such permissions are necessary before the receipt of the grant from the National Science Centre in Poland and are in accordance with the Law of the United Europe.

### Treatment protocol

Bitches were randomly assigned to either control (n = 5) or study group (n = 9). On the 19th and 20th day after LH peak, control bitches received saline solution (Baxter, Poland, 0.3ml/kg bw, subcutaneously), whereas study ones received RU534 (Alizine, Virbac, Carros, France, 10 mg/kg bw, subcutaneously).

### Blood samples

Blood samples (2 ml) were collected into dry tubes from the cephalic vein, daily starting from the 5th-7th day after the onset of external signs of the heat so as to detect LH peak (day 0)and then for 26 consecutive days. The LH peak (day 0) was identified on the basis of a certain P4 level (6.4–9.5 nmol/l) and the emergence of characteristic metestrus cells 8 days afterwards (foam, parabasal, and small intermediate) [3]. Blood samples were obtained before saline or RU534 treatment. Then blood samples were centrifuged (3000g for 15 min) and sera were stored at -20°C until assayed for P4 and total estrogens (E).

### Hormone assays

For quantitative determination of P4 and E commercial immunoenzymatic tests (Pointe Scientific, Poland) were used. Concentration of E was measured after extraction by ethyl acetate. Fluorescence was measured with a Pointe 2000 apparatus. Each analysis was repeated twice in each series. Efficiency of extraction oscillated between 92% and 99%. Assay sensitivity and intra-series errors for P4 were 0.05 ng/ml (0.8 nmol/l) and 8.0%, respectively, and for E 10 pg/ml (37 pmol/l) and 9.6%, respectively.

### Tissue collection

On day 26 after the estimated LH peak, control and study bitches, underwent elective ovariohysterectomy under general anesthesia induced by propofol (4 mg/kg bw) and maintained with isoflurane. Upon removal, the reproductive tracts were thoroughly washed with saline. The ovaries and parts of the wall of both uterine horns and bodies were excised and immediately placed in 10% neutral buffered formalin.

### Immunohistochemistry

For immunohistochemistry (IHC), paraffin-embedded sections were stuck onto glass slides covered with 2% Silane solution in acetone. After dewaxing in xylene and rehydrating in reverted alcohol dilution series the sections were boiled in 0.02 M citrate buffer of pH 6.0 in a microwave oven. After cooling, the sections were placed in 3% perhydrol, and then washed twice in distilled water. After 30 min incubation in 5% bovine serum albumin (Sigma Aldrich, Germany), the primary antibodies were used (diluted in 1% bovine serum): rabbit polyclonal anti-human E receptor alpha (against amino acids 2–185 of ERα, Santa Cruz Biotechnology, USA) diluted 1:100 and mouse monoclonal anti-human P4 receptor (clone PR10A9, Immunotech, France) antibodies were added in suitable dilutions according to the manufacturers’ instructions and were incubated in a humidified chamber for 60 minutes. Then, En Vision + System-HRP (Dako, Denmark) was employed to visualize the sections. In further steps the sections were washed in TRIS buffer and chromogen solution of 3,3’-diaminobenzidine (DAB), prepared according to the manufacturer’s instructions (Dako, Denmark), was added. In a final stage, the sections were carried along the alcohol series of increasing concentrations, xylene exposed and mounted with DPX medium (VWR, USA).

### Integrated optical density

Microscopic analysis was conducted under BX40 upright microscope (Olympus Poland, Warsaw, Poland) equipped with 5 MP DP50 3CCD peltier-cooled camera (Olympus Poland, Warsaw, Poland) and automated image analysis system. All microphotographs were collected using 40x air lens under reference lighting conditions for histology. Microphotographs were acquired using LifeView software (Olympus Poland, Warsaw, Poland) and automatically transferred to the Micro Image 4.0 (Olympus Poland, Warsaw, Poland) for analysis. Integrated optical density (IOD) of tissues with positive reaction was measured from ten randomly-chosen areas of interest (AOI) per each slide. The DAB-positive chromatic colour was sampled from 3x3 pixel area and automatically applied to all AOI. The average IOD from all 10 AOI was considered as an IOD measurement value per slide.

### Morphometry

All morphometric measurements were conducted using fully-automated BX40 upright microscope (Olympus Poland, Warsaw, Poland) equipped with motorised stage, high-resolution 12 MP monochromatic camera and Cell P (Olympus Poland, Warsaw, Poland) analysis software. Measurements were conducted under 10x, 20x, 40x or 100x air lenses under reference lighting conditions for histology. Diameter of ovarian follicles and corpora lutea, endometrial, myometrial and serosal thicknes, endometrial superficial epithelium height, endometrial glands epithelium height and endometrial glands sections area were measured.

### Statistical analysis

Results were given as means ± standard deviations (SD) and ranges in parentheses. A Shapiro-Wilk test was used to evaluate normality of data distribution and then either Student’s t-test for unpaired samples or Mann-Whitney U test were used to compare variables between groups. Equality of group variances was assessed with a Brown-Forsythe test. Student’s t-test for paired samples or Wilcoxon signed rank test were used to compare variables between the uterine horn and body in each group. All tests were two-tailed. The p-value below 0.05 was considered to indicate statistical significance. Statistical analyses were performed in Statistica 10.0 (StatSoft Inc., Poland).

## Results

The administration of RU534 did not cause any apparent side effects in the study bitches throughout the experiment, except for a temporary scratching of the injection site after administration. During next six months of follow-up no significant clinical signs were reported by the owners or observed in routine physical examinations.

### In vivo studies

**Fig 1:**
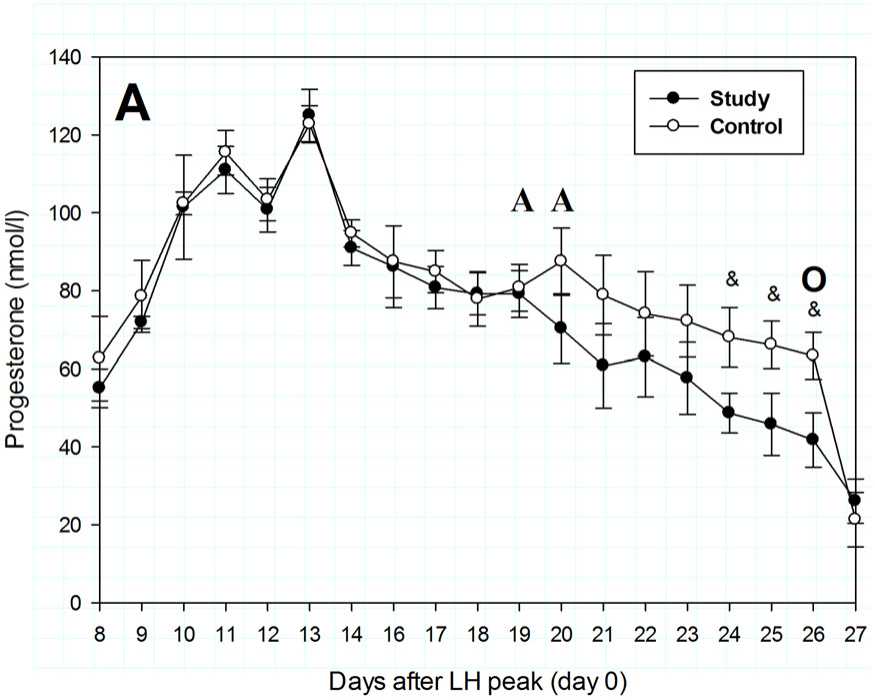
Serum progesterone concentrations in study (n = 9) and control (n = 5) bitches during the luteal phase, from days 8 to 27 after the estimated LH peak. The AA indicates days of treatment with aglepristone (10 mg/kg bw) or saline and O it is a day of ovariohysterectomy. Results are expressed as means ± SD. & and asterisks indicate values significantly different from controls (p<0.05 and p<0.01, respectively).

Before treatment, the mean serum P4 concentrations were 77.9 ± 7.0 nmol/l and 79.2 ± 6.0 nmol/l in the control and study group, respectively (Fig. 1). Functional corpora lutea (CL) were present at the beginning of the experiment in both groups, as indicated by basal circulating concentrations of plasma P4 (Fig. 1) and morphological examination. Five days after RU534 administration, days 24 through 27 after LH peak, P4 concentrations in the study group began to fall below P4 level in the control group (p<0.01) (Fig. 1).

**Fig 2:**
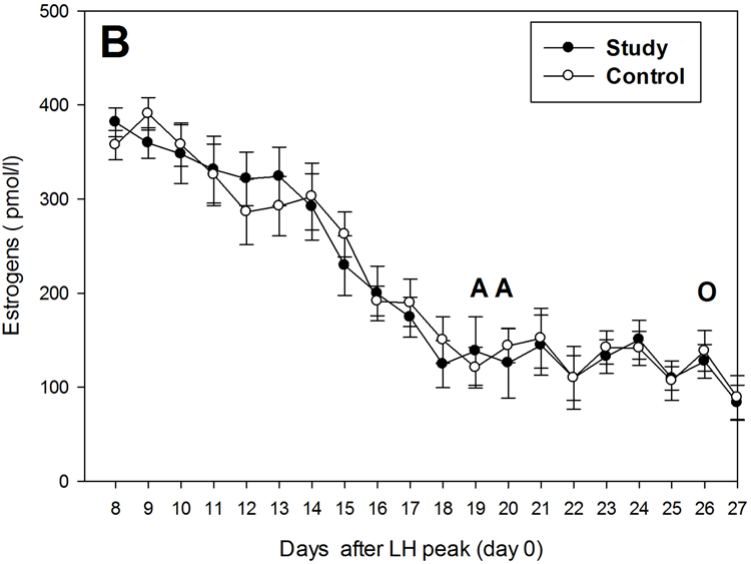
Serum estradiol concentrations in study (n = 9) and control (n = 5) bitches during the luteal phase, from days 8 to 27 after the estimated LH peak. The AA indicates days of treatment with aglepristone (10 mg/kg bw) or saline and O it is the day of castration. Results are expressed as means ± SD.

The mean serum E concentrations were relatively high before RU534 administration (124.4 ± 25.0 pmol/l), but gradually declined to 88.1–99.1 pmol/l on day 27 after LH peak, remaining at approximately the same levels thereafter (Fig. 2). Serum E levels did not differ in study and control bitches before and after RU534 administration for the entire study (Fig. 2).

### Ovarian morphology

**Fig 3:**
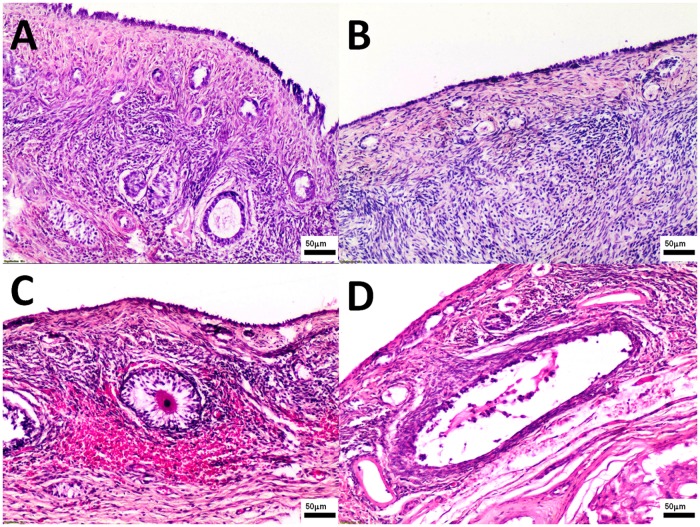
The ovary tissues were collected from control (Panel A, B) and study bitches (Panel C, D). In Fig. 3C, haemorrhages around primary/growing follicle. In Fig. 3D a big follicle. Microphotographs of ovary sections stained with H-E, original magnification 100x.

The pathomorphological findings in both groups were comparable (Fig. 3). Large follicles (a few in ovary) were found only in study bitches. There were no significant differences in the number of medium follicles (0.6 to 1.0 mm) between groups (Table 1). The CL were found in all study bitches (total 33, 1–6 per one individual) and all controls (21 total, 1–6 per one individual). No statistically significant differences were observed in the number of CL (0.6 to 1.0 mm and > 1.0 mm, respectively) between groups (Table 1). A few medium CL in the study group compared to none in the control group were found but this difference was not statistically significant. Haemorrhages and congestions in ovarian cortex were observed in six study and one control bitch (Fig. 3).

**Table 1:** Comparison of histomorphological changes in the structure of ovaries in study and control bitches.

Diameter (mm)	Study group (n = 9)	Control group (n = 5)	p-value
	Number [Mean±SD (min-max)]	Number [Mean±SD (min-max)]	
**F (<0.6)**	numerous	numerous	
**F (0.6–1.0)**	1.4±2.0 (0–6)	0.2±0.4 (0–1)	0.240
**F (>1.0)**	0.7±1.3 (0–3)	0	0.518
**CL (<0.6)**	0	0	
**CL (0.6–1.0)**	0.7±1.0 (0–3)	0	0.364
**CL (>1.0)**	6.5±2.4 (4–10)	4.8±1.5 (3–7)	0.190

F–ovarian follicles, CL–corpora lutea

### Uterine morphology

#### a. histology

**Fig 4:**
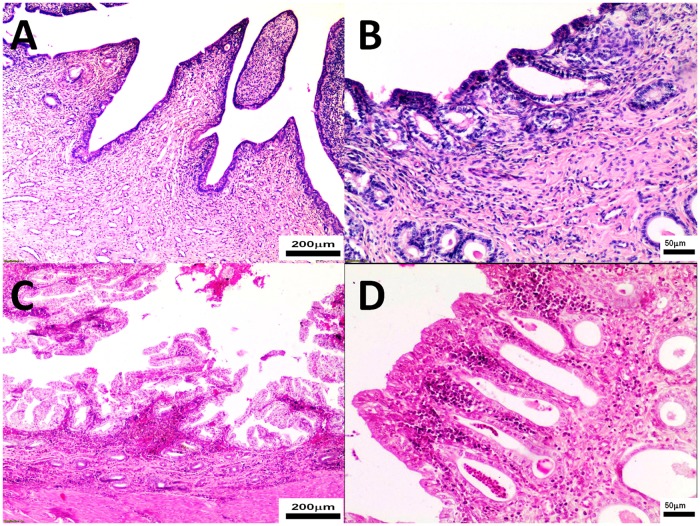
The uterine horns were collected from control (Panel A, B) and study bitches (Panel C, D). In Fig. 4C, endometrial hyperplasia, dilated glands, inflammatory cells and haemorrhages in the stroma. In Fig. 4D, dilated gland lined partially by epithelium with vacuolated cytoplasm, lymphocytes within the stroma were present. Microphotographs of uterine horn sections stained with H-E, original magnification 4A, 4C 40x; 4B, 4D 200x.

**Fig 5:**
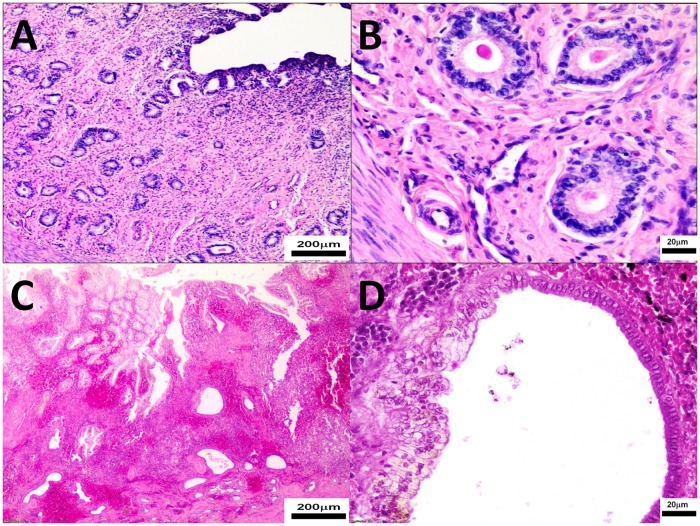
The uterine bodies were collected from control (Panel A, B) and study bitches (Panel C, D). In Fig. 5C, villous folds lined by endometrial epithelium with vacuolated cytoplasm. In Fig. 5B, mild mononuclear immune cell infiltration in endometrium. In Fig. 5D, endometrial hyperplasia associated with dense mononuclear immune cell infiltrate. Microphotographs of uterine bodies sections stained with H-E, original magnification 5A, 5C 400x; 5B, 5D 100x.

Microscopic appearance of the uterine horns varied in groups. In 6 bitches from the study group, mild to moderate inflammatory infiltration (made up mainly of mononuclear cells) was observed (Fig. 4). Moreover, in the study group microscopic appearance of uterine horns was comparable to uterine bodies, however the inflammatory process (infiltration of inflammatory cells, haemorrhages) observed in the study group uterine horns was more intensive than in uterine bodies (Figs. 4 and 5). Uterine horns and bodies were similar within the control group, however endometritis was milder compared to the study group. In all study bitches endometrium haemorrhages and blood congestion (of various intensity) were noted, but in 3 of them mainly in the superficial part (Figs. 4 and 5). In 2 cases microscopic appearance might imply presence of pseudo-placentational endometrial hyperplasia (PEH). In 5 bitches endometrial haemorrhages, located mainly in the superficial part, and brown granulation in epithelial cells (probably hemosiderin), unaccompanied by the inflammatory process, were evident (Fig. 4). Furthermore, moderate infiltration of mononuclear immune cells was observed around blood vessels of myometrium (4/9). Cross-sections of endometrial glands were markedly wider compared to the control group. Mild to moderate inflammatory infiltration composed mainly of mononuclear cells could be observed in 3 bitches from the control group, however, in general, inflammatory reaction was substantially milder than in the study group. In two study bitches necrosis of epithelial cells, in four considerable hyperplasia of glandular epithelium and in another 2 bitches vacuolation of superficial epithelial cells (foam cells) were observed. In another case endometrial haemorrhages located mainly in the superficial part of endometrium, unaccompanied by any inflammatory infiltration, were found (Fig. 2).

Microscopic appearance of uterine bodies was also diverse in groups. In 5 study bitches mild to moderate inflammatory infiltration (composed mainly of mononuclear immune cells) around blood vessels of myometrium was observed (3/9) (Fig. 5). In 4 cases hemosiderin granulation in epithelial cells of the superficial part of endometrium without any inflammatory process was found. In two study bitches necrosis of epithelial cells and in 3 cases mild to considerable hyperplasia of glandular epithelium and cysts were found (Fig. 5). In the study group haemorrhages were seen mainly in the superficial part of endometrium (3/9). Intensive congestion of endometrium and myometrium was found in 3 cases. No haemorrhages were noted in the control group. Cross-sections of endometrial glands were also considerably wider in the study group. In the control group mild inflammatory infiltration could be observed only in two bitches and there were in general much fewer endometrial glands compared to the study group (Fig. 5).

#### b. morphometry

When comparing uterine horns with bodies no significant differences were observed in endometrial, myometrial, serosal and endometrial epithelium height in any of groups (Table 2). Neither were any significant differences found in the myometrial and serosal thickness, endometrial epithelium height of uterine bodies and horns in groups (Table 2). Moreover, endometrial gland epithelium height and cross-section area of endometrial glands of uterine horns and endometrial thickness of bodies did not change after RU534 administration (Table 2). On the other hand, endometrial thickness of uterine horns in the study group was significantly higher than in controls (p<0.05) (Table 2).

**Table 2:** Comparison of histomorphological changes in the structure of uterine wall in study and control bitches.

Study group (n = 9)						Control group (n = 5)		
	Uterine horn		Uterine body			Uterine horn	Uterine body	
	Mean ± SD(min-max)	p- value ^1^	Mean ± SD(min-max)	p-value^2^	p-value^3^	Mean ± SD(min-max)	Mean ± SD(min-max)	p-value^4^
**ET(μm)**	1214.0±192.5 (971.4–1527.6)	0.008	1334.4±250.0 (835.4–1719.2)	<0.001	0.298	881.2±183.9 (735.0–1192.8)	811.0±98.9 (671.2–950.8)	0.486
**MT(μm)**	720.9±211.4 (424.0–1123.4)	<0.05	1873.8±716.5 (319.1–3102.8)	0.031	<0.001	487.4±33.7 (439.0–534.2)	2687.8±207.7 (2394.1–2981.5)	<0.001
**ST(μm)**	727.9±315.6 (120.7–1369.9)	0.213	518.2±420.8 (21.0–1223.3)	0.037	0.329	992.1±435.3 (588.6–1654.7)	672.3±299.5 (249.6–1095.1)	0.671
**ESEH(μm)**	15.1±3.9 (9.4–23.9)	0.158	19.5±5.3 (6.5–24.8)	0.851	0.033	18.3±3.7 (12.5–22.8)	18.9±4.9 2.0–25.8)	0.773
**EGEH(μm)**	11.9±2.3 (7.6–16.1)	0.808	10.2±2.9 (5.7–15.5)	-	0.284	11.6±0.9 10.8–13.2)	-	-
**EGA(μm^2^)**	18135.0±29019.7 (299.7–2781.3)	0.294	7494.5±4757.2 (83.0–15779.2)	-	0.282	3603.1±1960.1 (2015.2–6605.1)	-	-

ET–Endometrial thickness, MT–Myometrial thickness, ST–Serosal thickness, ESEH–Endometrial superficial epithelium height, EGEH–Endometrial glands epithelium height, EGA–Endometrial glands area,

p-value^1^–comparison of measurements of the uterine horns between study and control group,

p-value^2^–comparison of measurements of the uterine bodies between study and control group,

p-value^3^–comparison of measurements of the uterine horns and bodies in the study group,

p-value^4^–comparison of measurements of the uterine horns and bodies in the control group.

### Ovarian PR and ERα expression

**Fig 6:**
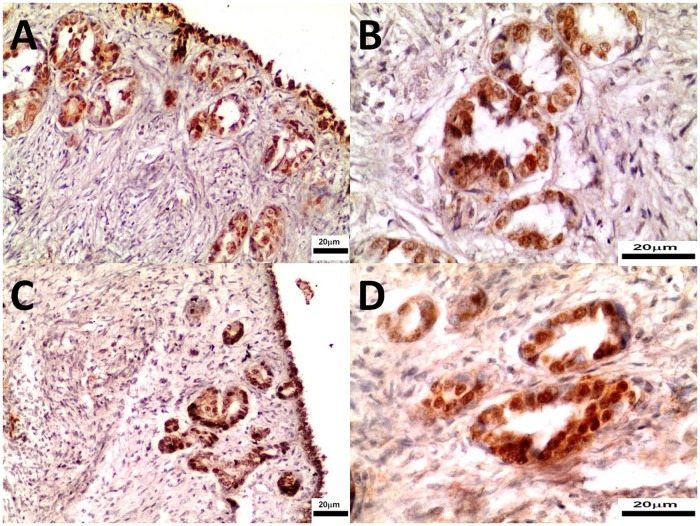
The immunohistochemistry of ERα in ovary tissues collected from control (Panel A, B) and study bitches (Panel C, D). In Figs. 6A-6D estrogen receptors in nuclei of epithelial cells of the ovary follicles and the ovarian superficial epithelium. Original magnification of microphotographs 6A, 6C 200x; 6B, 6D 400x.

**Fig 7:**
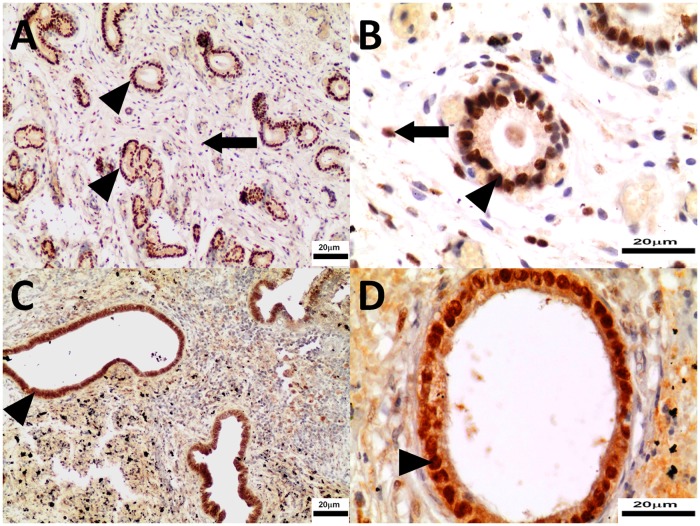
The immunohistochemistry of PR in ovary tissues collected from control (Panel A, B) and study bitches (Panel C, D). In Figs. 9C and 9D strong nuclear expression of progesterone receptors in epithelial cells of the ovary follicles and the ovarian superficial epithelium. Original magnification of microphotographs 9A, 9C 100x; 9B, 9D 400x.

Progesterone receptor and α-estrogen receptor expression were detected in ovaries of both control and study mid-luteal phase bitches (Figs. 6 and 7). On day 26 aglepristone treatment did not change PR and ERα expression (Table 3).

**Table 3:** Comparison of changes in expression of progesterone receptors (PR) and α-estrogen receptors (ERα) in the ovarian tissue in study and control bitches.

Receptors	Study group (n = 9)	Control group (n = 5)	p-value
	IOD [Mean ±SD (min-max)]	IOD [Mean ±SD (min-max)]	
**PR**	475.8±284.7 (164.8–1045.5)	291.1±228.1 (154.0–693.0)	0.212
**ERα**	818.5±211.2 (608.0–1276.8)	714.3±344.6 (322.0–1062.3)	0.587

PR–progesterone receptors, ERα – α estrogen receptors, IOD–integrated optical density.

### Uterine PR and ERα expression

**Fig 8:**
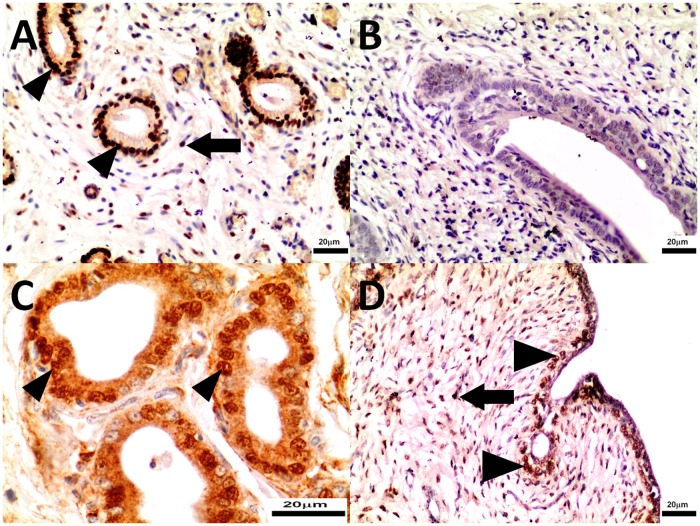
The immunohistochemistry of ERα in uterine bodies collected from control (Panel A, B) and study bitches (Panel C, D). In Figs. 8A, 8C and 8D estrogen receptors in nuclei of epithelial cell of endometrium (arrowheads), and in Figs. 8A and 8D estrogen receptors in nuclei of stromal cell (arrows). Original magnification of microphotographs 8A 200x, 8C 400x; 8B, 8D 200x.

**Fig 9:**
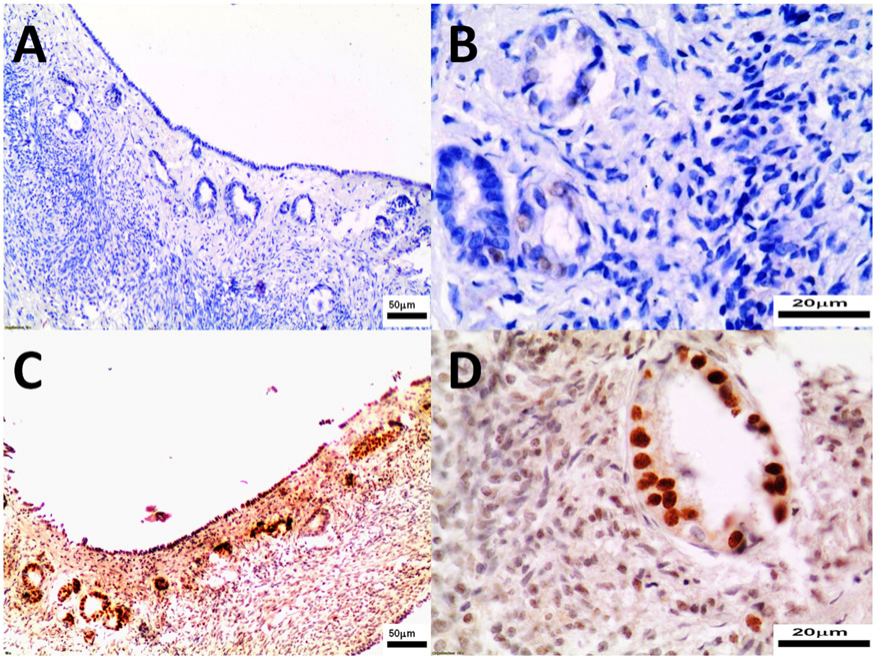
The immunohistochemistry of ERα in uterine horns collected from control (Panel A, B) and study bitches (Panel C, D). In Figs. 7C and 7D the expression of estrogen receptors in nuclei of epithelial cells of endometrium (arrowheads), in Figs. 7A and 7B expression of estrogen receptors in nuclei of stromal cells (arrow). Original magnification of microphotographs 7A, 7C 200x; 7B, 7D 400x.

**Fig 10:**
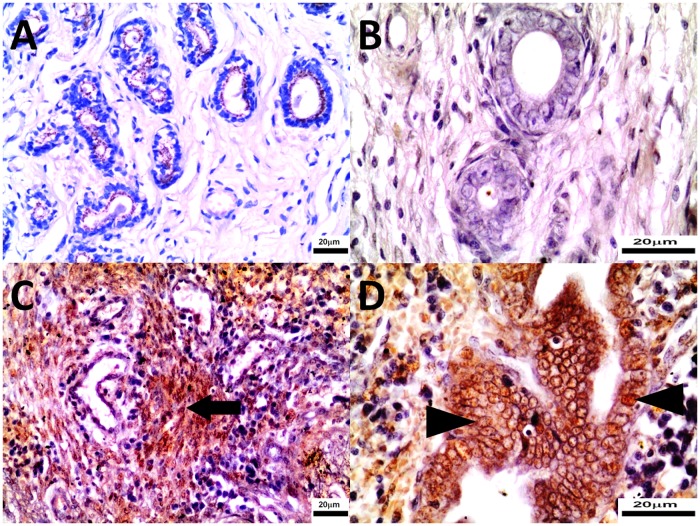
The immunohistochemistry of PR in uterine horns collected from control (Panel A, B) and study bitches (Panel C, D). In Fig. 10C progesterone receptors in nuclei of stromal cells (arrows), and in Fig. 10D progesterone receptors in nuclei of epithelial cells of uterine glands (arrowheads). Original magnification of microphotographs 10A, 10C 200x; 10B, 10D 400x.

**Fig 11:**
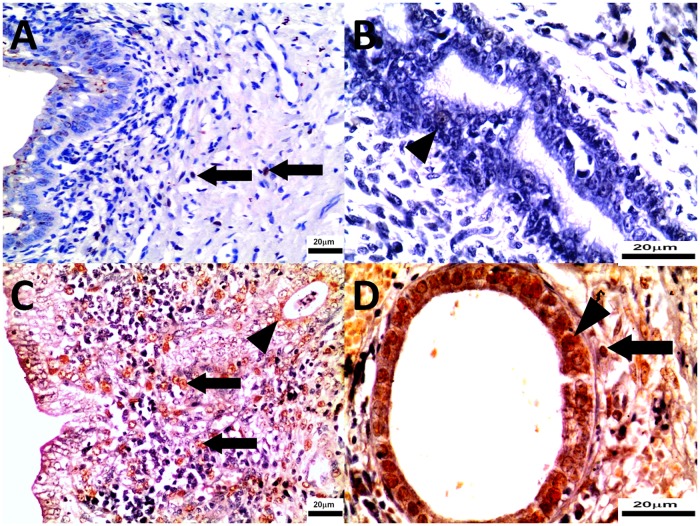
The immunohistochemistry of PR in uterine bodies collected from control (Panel A, B) and study bitches (Panel C, D). PR immunoreaction in nuclei of stromal cells (arrows) and epithelial cells of uterine glands (arrowheads). Original magnification of microphotographs 11A, 11C 200x; 11B, 11D 400x.

Progesterone receptor and α-estrogen receptor expression were detected in endometrium without surface epithelium (EwSE) and myometrium of both control and study mid-luteal phase bitches (Figs. 8, 9, 10, 11). There was no expression observed in serosa. Aglepristone increased (p<0.01) PR expression in endometrial epithelium and EwSE of uterine horns while it did not change expression in endometrial epithelium and EwSE of uterine bodies (Table 4). The expression of PR in myometrium remained unchanged in uterine horns, however it increased significantly in uterine bodies (p<0.05) (Table 4). Aglepristone therapy did not affect the expression of ERα in endometrial epithelium and myometrium of both uterine bodies and horns. Neither did the expression of ERα in EwSE of uterine bodies differ between groups. However, significant differences were observed in ERα expression in EwSE of uterine horns (two-fold greater than in the control group, p<0.05).

**Table 4:** Comparison of changes in expression of progesterone receptors (PR) and α-estrogen receptors (ERα) in uterine wall in study and control bitches.

Study group (n = 9)						Control group (n = 5)		
	Uterine horn		Uterine body			Uterine horn	Uterine body	
	IOD Mean ± SD(min-max)	p- value^1^	IOD Mean ± SD(min-max)	p-value^2^	p-value^3^	IOD Mean ± SD(min-max)	IOD Mean ± SD(min-max)	p-value^4^
**ESE–PR**	5521.1±6273.9 (313.9–16738.0)	<0.05	1699.9±1248.9 (375.0–4131.6)	0.067	0.100	629.7±1064.7 (50.7–2527.6)	536.2±307.6 (255.0–926.6)	0.819
**EwSE- PR**	3153.4±1834.5 (810.1–6589.3)	<0.05	2312.0±1548.5 (365.3–5720.6)	0.127	0.253	606.6±422.1 (141.4–1130.9)	1019.3±1096.2 (358.7–2964.8)	0.375
**M- PR**	1134.1±661.1 (205.0–2369.6)	0.056	1571.3±827.5 (436.4–2913.5)	<0.01	<0.01	439.6±406.0 (49.9–1073.7)	423.0±138.3 (215.6–600.0)	0.903
**ESE–ER**α	17376.0±21522.6 (1366.9–70700.4)	0.304	16645.4±16229.1 (2260.0–56634.3)	0.214	0.934	6612.7±6605.8 (672.2–4091.2)	6614.4±6030.4 (779.6–16069.2)	0.999
**EwSE- ER**α	3840.6±1656.3 (1153.9–6725.6)	<0.05	3489.0±1811.9 (668.7–6694.0)	0.117	0.631	2063.2±794.2 (1436.9–3441.5)	2002.1±964.6 (1094.6–3488.2)	0.903
**M- ER**α	2050.5±816.2 (760.6–3253.7)	0.109	2513.9±812.1 (873.0–3630.2)	0.133	0.070	1343.8±524.4 (637.3–2080.3)	1807.2±732.6 (924.3–2547.8)	0.063

ESE-PR–Endometrial surface epithelium progesterone receptors, EwSE-PR- Endometrium without surface epithelium progesterone receptors, M-PR–Myometrium progesterone receptors, ESE-ERα –Endometrial surface epithelium α estrogen receptors, EwSE-ERα –Endometrium without surface epithelium α estrogen receptors, M-ERα –Myometrium α estrogen receptors,

p-value^1^–comparison of receptor expression in the uterine horns between study and control group,

p-value^2^–comparison of receptor expression in the uterine bodies between study and control group,

p-value^3^–comparison of receptor expression in the uterine horns and bodies in the study group,

p-value^4^–comparison of receptor expression in the uterine horns and bodies in the control group.

When comparing uterine horns and bodies there were no significant differences observed in ERα expression in endometrial epithelium, EwSE and myometrium of the study and control group separately (Table 4). Neither did PR expression in endometrial epithelium and EwSE differ. However, in the study group PR expression was higher in myometrium of uterine bodies compared to horns. No such difference could be observed in the control group (Table 4).

## Discussion

Aglepristone, the P4 antagonist, is used in clinical practice for treatment of pathologies caused by a relatively long exposure to high P4 levels, including cystic endometrial hyperplasia [4]. Peripheral blood P4 concentration in the first part of luteal phase was high and similar to those found by Polisca et al. [3]. A gradual decline of P4 concentration observed in dogs given RU534 parallels that of controls, suggesting that this antiprogestagen triggers an anticipated and physiological-like luteolytic process. In the present study, decrease in P4 concentration in the study group began on the second day after RU534 administration and on the fifth day P4 level became significantly lower compared to controls (Fig. 1).

Ovarian ERα and PR were found mainly in CL of both groups which is similar to results of Parillo et al. [5] in rabbits. According to our results there is no relationship between RU534 treatment and ERα and PR expression in ovaries. To the knowledge of the authors there is no literature describing the influence of RU534 on morphology and ERα and PR expression in canine ovaries. However, unlike in rabbits [5] we found a slight difference–a few medium CL in the study group compared to none in the control group. The difference is not statistically significant but it can be a clue to explain the mechanism of premature luteolysis which was not confirmed in previous studies [6].

Mifepristone (RU486), used for pregnancy termination in humans increases peripheral concentration of PGFM, a main metabolite of prostaglandin F2α [6,7]. Another study has suggested that early cessation of the luteal phase may be due to a decrease in LH pulse amplitude, LH pulse frequency and LH secretion during treatment with RU486. It has been speculated that premature luteolysis may result from the action of this antiprogestagen compound at the hypothalamic-pituitary level [6]. This might also be a plausible explanation for the decrease of P4 concentration after RU534 administration. Detailed studies of the effect of RU534 on the hypothalamic-pituitary-ovarian axis in dogs are lacking.

Progesterone inhibits synthesis of ERα and PR [8]. Decline in P4 concentration may have blocked this mechanism and indirectly led to higher expression of these receptors in our study. This, in turn, could have increased sensitivity of tissues to E circulating in bloodstream. Therefore, despite no difference in E concentration between groups was observed, changes typical for the estrus such as congestion, thickening and inflammatory reaction with mononuclear cell infiltration could be found in endometrium in the study group. This mechanism has not been so far described in dogs.

No significant differences in endometrium between horns and bodies were noticed within groups. On the other hand, thicker myometrium and higher PR expression in uterine bodies compared to horns could have resulted from different functions of these parts of the uterus during parturition. As far as the authors know no such observations have been described to date.

Significant thickening of endometrium of both horns and bodies and myometrium of horns was likely to come from concomitant activity of E and P4 together with the increased expression of their receptors. This type of mechanism develops when follicular cysts are present on the ovaries and high level of P4 in diestrus parallels increased E level which eventually leads to cystic endometrial hyperplasia. However, the mechanism of action of RU534 presented here which consists in intensification of acute inflammatory reaction with mononuclear immune cell infiltration in endometrium is likely to hasten healing process and shorten therapy of chronic endometritis in classical pyometra. Furthermore, these observations elucidate mechanisms responsible for preventing fertilized ovum from implantation and, as a consequence, for a contraceptive action of RU534.

## Conclusions

Aglepristone administration causes acute inflammatory reaction with mononuclear immune cell infiltration in endometrium. Furthermore, it increases PR expression in EwSE of uterine horns, endometrial epithelium, myometrium of uterine bodies as well as expression of ERα in EwSE of uterine horns. As far as the authors know this is the first study which describes the inflammatory effect developing in the uterus in response to RU534 administration and attempts to elucidate its mechanisms. Nevertheless the influence of RU534 on the hypothalamic-pituitary-ovarian axis and mechanisms of its action in bitches just after implantation warrant further investigation.
